# Brazilian Red Propolis and Its Active Constituent 7-O-methylvestitol Impair Early and Late Stages of *Toxoplasma gondii* Infection in Human Placental Models

**DOI:** 10.3390/microorganisms13081937

**Published:** 2025-08-20

**Authors:** Samuel Cota Teixeira, Guilherme de Souza, Natalia Carine Lima dos Santos, Rafael Martins de Oliveira, Nagela Bernadelli Sousa Silva, Joed Pires de Lima Junior, Alessandra Monteiro Rosini, Luana Carvalho Luz, Aryani Felixa Fajardo Martínez, Marcos Paulo Oliveira Almeida, Guilherme Vieira Faria, Rosiane Nascimento Alves, Angelica Oliveira Gomes, Maria Anita Lemos Vasconcelos Ambrosio, Rodrigo Cassio Sola Veneziani, Jairo Kenupp Bastos, José Roberto Mineo, Carlos Henrique Gomes Martins, Eloisa Amália Vieira Ferro, Bellisa Freitas Barbosa

**Affiliations:** 1 Laboratory of Immunophysiology of Reproduction, Institute of Biomedical Science, Universidade Federal de Uberlândia, Campus Umuarama, Av. Pará, 1720, Uberlândia 38405-318, MG, Brazil; guisbio@hotmail.com (G.d.S.); carine.natalia@ufu.br (N.C.L.d.S.); rafael_martinso@hotmail.com (R.M.d.O.); joedjunior07@gmail.com (J.P.d.L.J.); monteirorosini_alessandra@hotmail.com (A.M.R.); luanacarvalholuz.28@gmail.com (L.C.L.); felixafajardo08@gmail.com (A.F.F.M.); marcospaulooliveiraalmeida@hotmail.com (M.P.O.A.); guilherme.vieira@ufu.br (G.V.F.); eloisa.ferro@ufu.br (E.A.V.F.); 2 Laboratory of Antimicrobial Testing, Institute of Biomedical Science, Universidade Federal de Uberlândia, Uberlândia 38405-319, MG, Brazil; nagela_bernadelli.mg@hotmail.com (N.B.S.S.); carlos.martins2@ufu.br (C.H.G.M.); 3 Department of Agricultural and Natural Science, Universidade do Estado de Minas Gerais, Ituiutaba 38302-192, MG, Brazil; rosiane.alves@uemg.br; 4 Institute of Natural and Biological Sciences, Universidade Federal do Triângulo Mineiro, Uberaba 38025-015, MG, Brazil; angelica.gomes@uftm.edu.br; 5 Nucleus of Research in Technological and Exact Sciences, Universidade de Franca, Franca 14404-600, SP, Brazil; malvasconcelos@yahoo.com.br (M.A.L.V.A.); rodrigo.veneziani@unifran.edu.br (R.C.S.V.); 6 School of Pharmaceutical Sciences of Ribeirão Preto, Universidade de São Paulo, Ribeirão Preto 14040-903, SP, Brazil; jkbastos@fcfrp.usp.br; 7 Laboratory of Immunoparasitology, Institute of Biomedical Sciences, Universidade Federal de Uberlândia, Uberlândia 38400-902, MG, Brazil; jrmineo@ufu.br

**Keywords:** congenital toxoplasmosis, maternal–fetal interface, trophoblast, drug discovery, natural products, alternative treatment

## Abstract

*Toxoplasma gondii* is a globally distributed protozoan parasite and a major cause of congenital infections, particularly in South America. Current therapies for congenital toxoplasmosis are limited by toxicity, long treatment regimens, and suboptimal efficacy, highlighting the urgent need for safer and more effective alternatives. In this study, we evaluated the antiparasitic effects of crude ethanolic extract of Brazilian Red Propolis (BRP) and its isolated compounds, focusing on 7-O-methylvestitol, in human trophoblast (BeWo) cells and third-trimester placental explants. Both BRP and 7-O-methylvestitol significantly reduced *T. gondii* adhesion, invasion, and intracellular replication, without compromising host cell viability. Ultrastructural analyses revealed irreversible parasite damage, and cytokine profiling demonstrated immunomodulatory effects, with enhanced production of interleukin (IL)-6, IL-8, and macrophage migration inhibitory factor (MIF) in BeWo cells and downregulation of IL-6, MIF, and tumor Necrosis Factor (TNF) in infected placental villi. Notably, 7-O-methylvestitol reproduced and, in some assays, surpassed the antiparasitic activity of BRP, suggesting it as a key bioactive constituent responsible for the therapeutic potential of the extract. These findings support the identification of 7-O-methylvestitol as a promising lead compound for structure-based drug design and repositioning strategies, advancing the development of novel, safe, and targeted therapies against congenital toxoplasmosis.

## 1. Introduction

Toxoplasmosis is a zoonotic disease caused by the protozoan parasite *Toxoplasma gondii*, with high prevalence worldwide [1]. Different strains of *T. gondii* have been identified, with variations in their virulence and pathogenicity. In South America, the strains show greater genetic variability and are more virulent, increasing the severity of the disease [2]. It is estimated that 30% of the global population is infected with *T. gondii*, representing a significant public health concern, particularly among immunocompromised individuals and pregnant women. Infection during pregnancy may lead to adverse outcomes such as spontaneous abortion, stillbirth, or the development of congenital toxoplasmosis [3].

Congenital toxoplasmosis is a serious health problem. The global incidence rate of congenital infection is around 1.5 cases/1000 live births, being more frequent in South America, in some countries of the Middle East, and in low-income countries [2]. Vertical transmission occurs more frequently when the infection is primary and varies according to gestational age [4]. The protective function of the placenta is most effective and critically important during the first trimester of pregnancy, with vertical transmission of the parasite occurring in up to 10% of cases of gestational toxoplasmosis. However, as placental vascularization and permeability increase in the second and third trimesters, the risk of transmission rises significantly, reaching 60–70% of cases. In such instances, infected neonates may exhibit severe clinical manifestations, including neurological impairments and ocular lesions [2].

Despite the severity of toxoplasmosis, its control still relies primarily on preventive measures, as no fully effective or universally adequate treatment is currently available. The standard first-line therapy consists of a combination of pyrimethamine and sulfadiazine or, in some cases, pyrimethamine with clindamycin, azithromycin, or trimethoprim-sulfamethoxazole. However, prolonged treatment duration, limited drug efficacy, and a high incidence of adverse effects often lead to poor adherence and recurrence of infection [5]. Therefore, the use of natural products represents a promising therapeutic alternative for the treatment of toxoplasmosis. In addition to their largely unexplored potential, natural compounds exhibit greater chemical diversity, structural complexity, and molecular rigidity when compared to synthetic molecules, which may enhance their efficacy against *T. gondii* [6].

One natural product that has gained considerable attention due to its diverse biological properties is Brazilian Red Propolis, a resinous substance derived from *Dalbergia ecastophyllum*. This plant species is frequently visited by *Apis mellifera* bees, which collect red exudates from its trunk and mix them with salivary secretions, resulting in the formation of red propolis [7,8]. The chemical composition of Brazilian Red Propolis is unique, composed of different isoflavonoids, such as vestitol, neovestitol, among others, which have several biological properties such as antimicrobial, anti-inflammatory, and antiparasitic [9,10,11]. However, it is important to notice that propolis’ chemical composition may vary due to several factors, such as seasonality, light exposure, altitude, local flora, and the bee species responsible for collection [12,13]. Despite its recognized pharmacological potential, the antiparasitic activity of Brazilian Red Propolis has not yet been investigated against *T. gondii*, especially in models of the human maternal–fetal interface.

In the human placenta, *T. gondii* has been shown to infect and replicate within cytotrophoblasts, syncytiotrophoblasts, endothelial cells, fibroblasts, and Hofbauer cells, including those located in the terminal chorionic villi. The parasite’s intracellular cycle in placental cells follows the same conserved sequence observed in other tissues, involving adhesion, invasion, and replication. Initially, tachyzoites adhere to host cell membranes through SAG and MIC proteins, mediating attachment and orientation. Subsequently, the parasite actively invades the host cell by forming a parasitophorous vacuole (PV), a process dependent on rhoptry and microneme secretory proteins. Inside the PV, *T. gondii* evades lysosomal degradation and replicates by binary fission, following a lytic cycle typical of its interaction with other host cell types. Studies have demonstrated that placental susceptibility to *T. gondii* infection varies according to cell type, gestational age, and immune status. While the syncytiotrophoblast has traditionally been considered more resistant to infection, recent evidence suggests that even this layer can be compromised, particularly under inflammatory conditions. Cells in the terminal chorionic villi, including stromal and endothelial components, have been shown to support tachyzoite replication, making them a relevant ex vivo model for studying congenital transmission mechanisms [14,15,16,17].

Red propolis has been shown to exhibit potent immunomodulatory effects. Its bioactive compounds, particularly isoflavonoids, flavonoids, and phenolic acids, can modulate both innate and adaptive immune responses by regulating cytokine production, inhibiting pro-inflammatory mediators, and enhancing antioxidant defenses [8,10,12]. In congenital toxoplasmosis, maternal infection is associated with elevated levels of pro-inflammatory cytokines, such as IL-6, IL-12, and IL-8, which may indicate an inflammatory placental environment capable of facilitating parasite transmission and placental damage. Simultaneously, the anti-inflammatory cytokine IL-10 plays a crucial role in modulating this response, helping to protect fetal tissues from excessive inflammation and promoting immune homeostasis at the maternal–fetal interface [18,19].

Therefore, the objective of the present study was to investigate the antiparasitic action of the crude ethanolic extract of Brazilian Red Propolis (BRP) and its isolated compounds (i.e., 7-O-methylvestitol, medicarpin, vestitol, and neovestitol) against *T. gondii* infection through two distinct experimental models: an in vitro model of human trophoblastic cells (BeWo cells) and an ex vivo model using human villous explants from the third trimester of pregnancy. These models are widely used in studies about congenital and gestational toxoplasmosis [20,21,22].

## 2. Materials and Methods

### 2.1. Cell Culture and Parasite Maintenance

Human trophoblast cells (BeWo lineage) were obtained from the American Type Culture Collection (CCL-98TM, ATCC, Manassas, VA, USA) and cultured in Roswell Park Memorial Institute (RPMI)-1640 medium (Cultilab, Campinas, SP, Brazil) enriched with 100 U/mL penicillin (Sigma Chemical Co., St. Louis, MO, USA), 100 μg/mL streptomycin (Sigma), and 10% heat-inactivated fetal bovine serum (FBS) (Cultilab). The cultures were maintained at 37 °C in a humidified environment with 5% CO_2_. In line with protocol number 13/2012, the Ethics Committee at the Universidade Federal de Uberlândia, MG, Brazil, confirms that no ethical approval is required for commercially acquired cell lines.

*Toxoplasma gondii* tachyzoites of the highly virulent RH strain (2F1 clone), which constitutively express the β-galactosidase gene, were maintained by continuous serial passages in BeWo cells. These parasites were cultured in RPMI 1640 medium supplemented with 2% FBS, 100 U/mL penicillin, and 100 μg/mL streptomycin, with incubation at 37 °C and 5% CO_2_.

### 2.2. Obtaining and Characterization of Crude Hydroalcoholic Extract of Brazilian Red Propolis (BRP) and Isolated Compounds

Brazilian Red Propolis (registered at the National System for the Management of Genetic Heritage and Associated Traditional Knowledge—SISGEN as AF234D8) was obtained from the Association of Beekeepers of Canavieiras (Cooperativa de Apicultores de Canavieiras, COAPER, Bahia, Brazil) from March 2019 to February 2020, and all chemical standards, including the compounds investigated herein (7-O-methylvestitol, medicarpin, vestitol, and neovestitol), were provided by Prof. Dr. Jairo Kenupp Bastos (School of Pharmaceutical Sciences of Ribeirão Preto, University of São Paulo, São Paulo, SP, Brazil). To obtain BRP, Brazilian Red Propolis was submitted to dynamic maceration at 30 °C and 120 rpm using a shaker incubator (INNOVA 4300, New Brunswick Scientific, Enfield, CT, USA) with 70% hydroalcoholic ethanol solution. The extract was then concentrated under vacuum using a rotary evaporator and lyophilized to complete dryness [23]. Chemical characterization of BRP was performed through HPLC analyses in comparison with authentic standards as described in Neto et al. (2022) [23].

### 2.3. Host Cell Viability

The toxicity of compounds was assessed using BeWo cells through the MTT colorimetric assay, following a previously published protocol [24]. BeWo cells (3 × 10^4^ cells/well/200 µL) were cultured in 96-well microplates for 24 h in RPMI 1640 medium supplemented with 10% FBS at 37 °C and 5% CO_2_. After incubation, the cells were treated with twofold serial dilutions of BRP, vestitol, neovestitol, 7-O-methylvestitol, and medicarpin (ranging from 256 to 4 µg/mL) for 24 h. In parallel, cells were treated with 0.6% DMSO, equivalent to the percentage used in the highest concentration tested (256 µg/mL). Untreated cells, exposed only to the culture medium, were used as a positive control for cell viability, considered 100% viable. After treatment, supernatants were removed, and the cells were incubated with MTT (5 mg/mL in RPMI medium, Sigma) for 4 h at 37 °C and 5% CO_2_. Formazan crystals were solubilized using a solution containing 10% SDS and 50% N,N-dimethylformamide for 4 h, and optical densities were measured at 570 nm using a plate reader. Cell viability was expressed as a percentage relative to untreated cells [20], and CC_50_ was obtained.

### 2.4. T. gondii Intracellular Proliferation

To assess the effect of BRP and isolated compounds on the *T. gondii* intracellular proliferation, a β-galactosidase assay was conducted following a previously established protocol [20]. BeWo cells (3 × 10^4^ cells/well/200 µL) were seeded in 96-well microplates for 24 h in RPMI 1640 medium supplemented with 10% FBS at 37 °C and 5% CO_2_. After, cells were infected with *T. gondii* tachyzoites at a multiplicity of infection (MOI) of 3:1 (three parasites per cell) in a culture medium containing 2% FBS and incubated for 3 h at 37 °C and 5% CO_2_. Afterward, the medium was discarded, and non-invaded parasites were carefully removed by rinsing with 1× PBS. The cells were then treated with BRP, vestitol, neovestitol, 7-O-methylvestitol, and medicarpin (ranging from 256 to 4 µg/mL) for 24 h or culture medium only (untreated cells) at 37 °C and 5% CO_2_. In parallel, the gold standard treatment with the association of sulfadiazine (200 μg/mL, Sigma) and pyrimethamine (8 μg/mL, Sigma) (SP). Parasite proliferation was quantified using a β-galactosidase assay with the chlorophenol red-β-D-galactopyranoside substrate (CPRG; Roche Diagnostics, Mannheim, Germany), and absorbance was measured at 570 nm. A standard curve of free tachyzoites (ranging from 1 × 10^6^ to 15.625 × 10^3^ parasites) was used to calculate the number of tachyzoites. Results were expressed as a percentage of *T. gondii* proliferation relative to untreated cells, considered to represent 100% parasite growth. Dose-response inhibition curves (Log (inhibitor) vs. normalized response—variable slope) were calculated. The therapeutic index (TI) was determined based on the CC_50_ BeWo cells/IC_50_ *T. gondii* ratio.

### 2.5. Transmission Electron Microscopy (TEM)

To assess the ultrastructure of intracellular *T. gondii* tachyzoites, BeWo cells (1 × 10^6^ cells/well/2000 µL) were seeded in 6-well microplates for 24 h in RPMI 1640 medium supplemented with 10% FBS at 37 °C and 5% CO_2_. After, cells were infected with *T. gondii* tachyzoites at an MOI of 3:1. After 3 h, cells were carefully rinsed with 1× PBS and incubated for 24 h at 37 °C and 5% CO_2_ with BRP (64 µg/mL), 7-O-methylvestitol (64 µg/mL), SP (200 + 8 µg/mL), or culture medium only (untreated group). BeWo cells were harvested, fixed with Karnovsky solution containing 2% paraformaldehyde and glutaraldehyde in a 0.1 M sodium cacodylate buffer (pH 7.4) for 24 h, washed with 1× PBS, post-fixed for 1 h in 1% OsO_4_ in cacodylate buffer and processed as described previously [20], before examination with a transmission electron microscope (Hitachi, TM 3000, Tokyo, Japan).

### 2.6. Reversibility Assay

This assay was conducted to verify the possible reversibility of the treatment after its removal [20]. BeWo cells (3 × 10^4^ cells/well/200 µL) were seeded in 96-well microplates for 24 h in RPMI 1640 medium supplemented with 10% FBS at 37 °C and 5% CO_2_. After, the cells were infected with *T. gondii* tachyzoites at an MOI of 3:1 in a culture medium containing 2% FBS and incubated for 3 h at 37 °C and 5% CO_2_. Subsequently, the cells were rinsed with 1× PBS to remove non-internalized parasites and then treated with BRP (64 µg/mL), 7-O-methylvestitol (64 µg/mL), SP (200 + 8 μg/mL), or culture medium only (untreated cells) for 24 h at 37 °C and 5% CO_2_. After the treatment period, the parasite proliferation was quantified using a β-galactosidase assay, previously described, or rinsed and incubated with RPMI medium free of treatments for an additional 24 h, followed by another β-galactosidase assay. Results were expressed as a percentage of *T. gondii* proliferation relative to untreated cells, considered to represent 100% parasite growth.

To corroborate the reversibility data, we investigated whether the treatments of BeWo cells infected with *T. gondii* tachyzoites would interfere with the ability of these parasites to invade and proliferate inside new fresh cells, as previously published protocols [20]. In brief, BeWo cells (1 × 10^6^ cells/2000 μL/well) were seeded in 6-well microplates, infected with parasites at an MOI of 3:1 for 3 h at 37 °C and 5% CO_2_, and treated for 24 h as mentioned above. Following, the intracellular parasites were obtained from treated BeWo cells by multiple passages through a 21- and 26-gauge needle and then allowed to infect (MOI 3:1) BeWo cells previously seeded in 96-well microplates (3 × 10^4^ cells/200 μL/well). After 3 and 24 h, the percentages of parasite invasion (% of *T. gondii* invasion) and parasite proliferation (% of *T. gondii* proliferation), respectively, were calculated using β-galactosidase assay.

### 2.7. Parasite Viability: Trypan Blue Staining and Scanning Electron Microscopy (SEM) Analysis

To investigate the direct effect of the treatments on *T. gondii*, we used two complementary methodologies: light microscopy and scanning electron microscopy (SEM). Briefly, 1 × 10^6^ free tachyzoites (RH strain, 2F1 clone) were added to microtubes in the presence of BRP (64 µg/mL), 7-O-methylvestitol (64 µg/mL), SP (200 + 8 μg/mL), or culture medium only (untreated parasites) for 1 h at 37 °C and 5% CO_2_. Afterwards, treatments were removed, and the parasites were submitted to two distinct experimental procedures: (1) parasites were incubated with trypan blue, counted in Neubauer chamber under a light microscope according to the following parameters: parasites with typical morphology (arched-shaped body), rounded, or dead (indicated by trypan blue staining). The results are presented as the percentage of parasites that fit into each parameter. (2) Tachyzoites were fixed in Karnovsky’s solution (2% glutaraldehyde and 2% paraformaldehyde) for 3 h. After fixation, the samples were rinsed with potassium cacodylate buffer and treated with 1% osmium tetroxide (OsO_4_) for 1 h. The parasites were then concentrated, placed onto circular coverslips (13 mm), and left to dry overnight at room temperature. Dehydration was carried out using a graded ethanol series (50%, 70%, 80%, 90%, 95%, and 100%). The samples were coated with a thin layer of gold and examined using a scanning electron microscope (Tescan Vega-3 LMU, Brno, Czech Republic).

### 2.8. Adhesion Assay of Pre-Treated T. gondii to BeWo Cells

In order to assess the direct impact of BRP and 7-O-methylvestitol on the early steps of parasite infection, parasites were pre-treated prior to infection and then proceeded with an adhesion assay. Briefly, BeWo cells (1 × 10^5^ cells/well/500 µL) were seeded in 24-well microplates containing 13 mm circular coverslips for 24 h at 37 °C and 5% CO_2_. After, the adhered cells were fixed with 4% paraformaldehyde (PFA) for 30 min at room temperature and then washed with 1× PBS. In parallel, *T. gondii* tachyzoites at an MOI of 3:1 were pre-incubated with BRP (64 µg/mL), 7-O-methylvestitol (64 µg/mL), SP (200 + 8 μg/mL), or culture medium only (untreated parasites) for 1 h at 37 °C and 5% CO_2_. Afterwards, treatments were removed, and the parasites were allowed to interact with fixed BeWo cells for 3 h at 37 °C and 5% CO_2_.

The non-adherent parasites were removed by washing with 1× PBS, and the adherent parasites were fixed under the same conditions as mentioned above. The coverslips were incubated with rabbit polyclonal primary anti-*T. gondii* antibody (Abcam, Waltham, MA, USA; #20530) [diluted 1:500 in PGN (PBS containing 0.25% gelatin)] for 17 h at 4 °C. Next, the coverslips were carefully rinsed with 1× PBS and then incubated with Alexa Fluor 488-conjugated anti-rabbit IgG (Invitrogen, #A11008, Waltham, MA, USA) (1:500), tetramethylrhodamine isothiocyanate (TRITC)-conjugated phalloidin (Sigma, P1951) (1:50), and TO-PRO-3 Iodide (Life Technologies, Waltham, MA, USA) (1:500), all diluted in PGN + saponin for 1 h at room temperature to label tachyzoites of *T. gondii*, F-actin, and nuclei, respectively. The coverslips were then mounted onto glass slides, and the samples were examined using a confocal fluorescence microscope (Zeiss LSM 510 Meta, Jena, Germany) equipped with an inverted microscope (Zeiss Axiovert 200 M). The total number of adhered parasites per cell in a total of 20 fields chosen randomly.

### 2.9. Invasion and Attachment Assay of Pre-Treated T. gondii to BeWo Cells

To gain insights into the impact of BRP and 7-O-methylvestitol on the early stages of *T. gondii* infection, we further proceed with a red/green differential antibody staining assay [20], with minor procedural modifications. BeWo cells (1 × 10^5^ cells/well/500 µL) were seeded in 24-well microplates containing 13 mm circular coverslips for 24 h at 37 °C and 5% CO_2_. *T. gondii* tachyzoites at an MOI of 3:1 were pre-incubated with BRP (64 µg/mL), 7-O-methylvestitol (64 µg/mL), SP (200 + 8 μg/mL), or culture medium only (untreated parasites) for 1 h at 37 °C and 5% CO_2_. After treatment removal, parasites were allowed to interact with previously adhered BeWo cells (1 × 10^5^ cells/24-well/500 µL) in 13 mm circular coverslips for 3 h at 37 °C and 5% CO_2_. After, non-invaded parasites were carefully removed with 1× PBS, and the cells were fixed with 4% PFA for 12 min at room temperature and incubated for 1 h with a rabbit polyclonal anti-*T. gondii* primary antibody (Abcam #20530, diluted 1:500 in PBS containing 0.25% gelatin [PGN]), followed by a secondary antibody Alexa Fluor 594-conjugated anti-rabbit IgG (Invitrogen #A11012, 1:500 in PGN). Next, cells were permeabilized using PGN with 0.01% saponin and incubated again with the same primary antibody (1:500), followed by Alexa Fluor 488-conjugated anti-rabbit IgG (Invitrogen #A11008, 1:500) and the nuclear stain TO-PRO-3 Iodide (Life Technologies, 1:500). Finally, coverslips were mounted on glass slides, and samples were visualized using confocal fluorescence microscopy (Zeiss LSM 510 Meta) with an inverted microscope (Zeiss Axiovert 200 M). Quantification was performed by analyzing 20 randomly selected fields per coverslip, counting intracellular parasites (green^+^/red^−^) and adhered parasites [red^+^ or red^+^/green^+^ (yellow)]. The invasion ratio was calculated as the proportion of intracellular tachyzoites relative to the total number of parasites.

### 2.10. Invasion and Intracellular Proliferation of T. gondii Tachyzoites

To decipher the potential targets of BRP and 7-O-methylvestitol, we promoted the pre-treatment of *T. gondii* tachyzoites (1 h) or BeWo cells (24 h) and assessed parasite invasion and proliferation rates using β-galactosidase activity. In the first set of experiments, *T. gondii* tachyzoites (MOI 3:1) were incubated with BRP (64 µg/mL), 7-O-methylvestitol (64 µg/mL), SP (200 + 8 μg/mL), or culture medium only (untreated parasites) for 1 h at 37 °C and 5% CO_2_. After, the parasites were centrifuged and resuspended in RPMI medium supplemented with 2% FBS and then allowed to interact with previously adhered BeWo cells (3 × 10^4^ cells/96-well/200 µL) for 3 h at 37 °C and 5% CO_2_.

In the second set of experiments, BeWo cells (3 × 10^4^ cells/well/200 µL) seeded in 96-well microplates were treated with BRP (64 µg/mL), 7-O-methylvestitol (64 µg/mL), SP (200 + 8 μg/mL), or culture medium only (untreated cells) for 24 h at 37 °C and 5% CO_2_ prior to parasite infection. Subsequently, *T. gondii* tachyzoites at an MOI of 3:1 in a culture medium containing 2% FBS were allowed to invade (3 h) and proliferate (24 h) with previously treated BeWo cells. At the end of both assays, parasite load was quantified by measuring β-galactosidase activity, as mentioned above.

### 2.11. Measurement of Intracellular Reactive Oxygen Species (ROS)

The production of intracellular reactive oxygen species (ROS) was quantified in both infected and non-infected BeWo cells, using the ROS-specific probe 2’,7’-dichlorodihydrofluorescein diacetate (H2DCF-DA). Briefly, BeWo cells (3 × 10^4^ cells/well/200 µL) were seeded in a black 96-well microplates with clear bottoms (Costar REF# 3603, New York, NY, USA) for 24 h at 37 °C and 5% CO_2_ and then infected with *T. gondii* tachyzoites (MOI 1:3) for 3 h at 37 °C and 5% CO_2_. After, the cells were washed with 1× PBS to remove non-internalized parasites and treated with BRP (64 µg/mL), 7-O-methylvestitol (64 µg/mL), SP (200 + 8 µg/mL), or culture medium only (untreated cells) for 24 h at 37 °C and 5% CO_2_. Additionally, cells treated with 3.5% hydrogen peroxide (H_2_O_2_) diluted in 1× PBS for 30 min at room temperature were considered a positive control group of ROS production. Finally, the supernatant was collected for cytokine measurement, and the cells were rinsed with 1× PBS and incubated with H_2_DCF-DA (10 μM; diluted in 1× PBS containing 10% FBS) in the dark for 45 min at 37 °C and 5% CO_2_. The DCF fluorescence was measured using a spectrofluorometer at 475/500 nm (excitation/emission) (VersaMax, Molecular Devices, San Jose, CA, USA).

### 2.12. Human Placental Explant Culture

Ex vivo assays using third-trimester human placental explants were performed to further elucidate our in vitro data. Third-trimester placentas (36 to 40 weeks, N = 3) from cesarean deliveries of healthy pregnant women aged 18 to 45 years were kindly donated by the participants. Infectious or non-infectious diseases, such as Chagas disease, toxoplasmosis, leishmaniasis, diabetes, chronic hypertension, and preeclampsia, were used as exclusion criteria. After placenta collection at the Clinic Hospital from Universidade Federal de Uberlândia (HC-UFU/MG-Brazil), the organ was washed with 1× PBS to remove excess blood, and the placental cotyledons were dissected. Terminal chorionic villi containing about four to eight free tips (~10 mm^3^) were collected. The dissected explants were then incubated in 96-well microplates (one per well) containing RPMI supplemented with 10% FBS for 24 h at 37 °C and 5% CO_2_ for further viability and parasitic assays [20]. This study was conducted in accordance with relevant guidelines and regulations, and the experimental protocols were approved by the Ethics Committee of the Federal University of Uberlândia (UFU), MG, Brazil, under approval number 7407162, 31 January 2024.

### 2.13. Viability Assays of Human Placental Explants

To identify treatment concentrations with potential toxicity to placental explants, two methodologies were employed: histological analysis and quantification of lactate dehydrogenase (LDH) production. For viability analysis via LDH quantification, placental explants were collected and cultured as described above (see item 2.12) and treated or not with BRP and 7-O-methylvestitol at the three highest concentrations used in BeWo cells, i.e., 256, 128, and 64 µg/mL. Additionally, villous explants were also submitted to the conventional therapy with SP (150 + 200 μg/mL, respectively), as a baseline for comparison. In all conditions, villous explants were maintained under for 24 h at 37 °C and 5% CO_2_. After, the supernatant was collected for LDH quantification using a commercial kit, following the manufacturer’s specifications (Bioclin, Belo Horizonte, MG, Brazil). LDH levels were measured using a microplate reader set to 340 nm, and the data were expressed as LDH units per liter (U/L).

To corroborate the data obtained from the LDH assay, the highest treatment concentration (256 µg/mL) was further evaluated for toxicity through morphological analysis by histological methods. For this, villous explants previously treated with BRP, 7-O-methylvestitol (256 µg/mL), SP (150 + 200 μg/mL), or culture medium only (untreated explants) for 24 h were processed using histological methods and stained with hematoxylin and eosin (HE). Tissue integrity analysis was performed using a light microscope (Opton), and representative micrographs that illustrated the tissue architecture were obtained using a coupled camera (Leica ICC50 Camera, Leica Microsystems, Lane Cove West, NSW, Australia).

### 2.14. T. gondii Infection of Human Villous Explants and Treatments

To assess *T. gondii* proliferation ex vivo, placental explants were collected and cultured as previously described and then infected with *T. gondii* tachyzoites (1 × 10^6^ per well/200 µL) for 24 h at 37 °C and 5% CO_2_. After the incubation period, villi were washed with 1× PBS to remove non-internalized parasites and then treated with BRP (ranging from 256 to 64 µg/mL), 7-O-methylvestitol (ranging from 256 to 64 µg/mL), sulfadiazine + pyrimethamine (SP; 150 + 200 μg/mL, respectively), or culture medium only (untreated explants) for 24 h at 37 °C and 5% CO_2_. Finally, the supernatants and placental explants were collected and stored at −80 °C for later analysis of cytokine production, protein quantification, and β-galactosidase assay.

Protein quantification was performed using the Bradford method. Frozen villous explants were macerated in a solution containing radioimmunoprecipitation assay buffer (RIPA) [50 mM Tris-HCl, 150 mM NaCl, 1% Triton X-100, 1% (*w*/*v*) sodium deoxycholate, and 0.1% (*w*/*v*) SDS, pH 7.5] with a protease inhibitor cocktail (Complete, Roche Diagnostic, Mannheim, Germany). The homogenate was centrifuged, and the supernatant was collected to determine the total protein concentration using the Bradford reagent (Sigma).

For parasite quantification, the supernatant from the previously obtained homogenate was used for the β-galactosidase reaction. The quantity of *T. gondii* tachyzoites was normalized based on the total protein concentration (µg/mL) of each villous sample, as determined by the Bradford assay. Results were expressed as the number of parasites per µg of tissue. The intracellular proliferation of *T. gondii* in villous explants was represented as a percentage (% of *T. gondii* proliferation), with the number of tachyzoites calculated by comparison to a standard curve of free tachyzoites (ranging from 1 × 10^6^ to 15.625 × 10^3^ parasites). For controls, the number of tachyzoites was quantified in untreated, infected villous explants incubated with culture medium alone (negative control). This condition was considered 100% parasite proliferation. The number of parasites in each treatment condition was then expressed as a percentage of *T. gondii* proliferation relative to the negative control.

### 2.15. Cytokines Quantification

The levels of the human cytokines IL-6, IL-8, IL-10, MIF, and TNF released in culture supernatants, produced by BeWo cells or placental explants, cultured under the different experimental conditions, were measured using a double-antibody sandwich enzyme-linked immunosorbent assay (ELISA). This analysis was conducted following the manufacturer’s instructions (Duoset R&D Systems, Minneapolis, MN, USA, for MIF; OpTEIA, BD Bioscience, San Diego, CA, USA, for the other cytokines). Cytokine levels in placental explants were normalized by calculating the ratio of cytokine production (pg/mL) to the total protein content (µg/mL). For placental explants, concentrations were expressed as pg/mL per mg of tissue, while values for BeWo cells remained in pg/mL. The sensitivity thresholds for each cytokine, determined through standard curve analysis, were as follows: IL-6 (4.7 pg/mL), IL-8 (3.1 pg/mL), IL-10, MIF, and TNF (all 7.8 pg/mL).

### 2.16. Statistical Analysis

Statistical analyses and graph generation were performed using GraphPad Prism v. 9.0 (GraphPad Software, Inc., San Diego, CA, USA). Data are expressed as the mean ± standard error of the mean (SEM). Differences among multiple groups were assessed by using one-way ANOVA with Sidak’s multiple comparison post-test for parametric data or by Kruskal–Wallis test with Dunn’s multiple comparison post-test for non-parametric data. Data were considered statistically significant at *p* < 0.05. The data were obtained from three independent experiments with eight replicates, at least.

## 3. Results

### 3.1. BRP and Its Isolated Compounds Are Non-Toxic at Low Concentrations and Control T. gondii Replication in BeWo Cells

**Figure 1:**
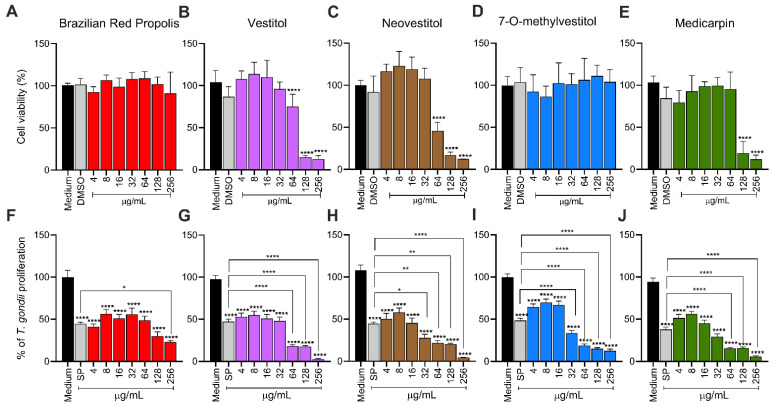
BRP and its derivatives are non-toxic at low concentrations and effectively control *T. gondii* proliferation in BeWo cells. (**A**–**E**) BeWo cells were exposed for 24 h to concentrations in twofold serial dilutions of either BRP, vestitol, neovestitol, 7-O-methylvestitol, and medicarpin (ranging from 4 to 256 µg/mL). In parallel, cells were treated with 0.6% DMSO, corresponding to the percentage used at the highest tested concentration. Untreated cells, exposed only to the culture medium, served as the positive control for cell viability and were considered 100% viable. (**F**–**J**) *T. gondii*-infected BeWo cells were exposed for 24 h to concentrations in twofold serial dilutions of either BRP, vestitol, neovestitol, 7-O-methylvestitol, and medicarpin (ranging from 4 to 256 µg/mL), a combination of sulfadiazine (200 μg/mL) plus pyrimethamine (8 μg/mL) (SP), or culture medium only (considered as 100% parasite proliferation). Intracellular parasite proliferation was assessed using the β-galactosidase assay and expressed as a percentage change relative to the control (% *T. gondii* proliferation). Data are shown as means ± standard error of the mean (SEM). Asterisks without brackets indicate comparisons versus the control group (black bar). Asterisks with brackets indicate comparisons between experimental groups. Differences were considered statistically significant when *p* < 0.05.

To evaluate the cytotoxicity of BRP, Vestiol, Neovestiol, 7-O-methylvestitol, and medicarpin, the MTT viability assay was performed. According to Figure 1, DMSO (0.6%; percentage used in the highest concentration of 256 µg/mL) treatments did not show toxicity for BeWo cells. The results for different concentrations of BRP and 7-O-methylvestitol showed no toxicity at any of the tested concentrations (Figure 1A,D). Vestiol and Neovestiol were toxic only at the highest concentrations (64, 128, and 256 µg/mL|**** *p* < 0.0001; Figure 1B,C), while medicarpin reduced cell viability only at concentrations of 128 and 256 µg/mL (**** *p* < 0.0001; Figure 1E) when compared to untreated BeWo cells (control group).

To assess the potential of BRP and its isolated compounds in controlling the intracellular proliferation of *T. gondii*, a β-galactosidase assay was performed. All compounds and concentrations assessed (ranging from 4 to 256 µg/mL) significantly reduced *T. gondii* proliferation in BeWo cells (**** *p* < 0.0001; Figure 1F–J) in relation to infected/untreated BeWo cells. As expected, the classical treatment with SP significantly reduced the growth of the parasite compared to the control group (**** *p* < 0.0001; Figure 1F–J). In comparison with the classical association with SP, BRP (256 μg/mL|* *p* < 0.05) (Figure 1F), vestitol (64 to 256 μg/mL|**** *p* < 0.0001) (Figure 1G), neovestitol (32 μg/mL|* *p* < 0.05; 64 and 128 μg/mL|** *p* < 0.01; 256 μg/mL|**** *p* < 0.0001) (Figure 1H), 7-O-methylvestitol (32 to 256 μg/mL|**** *p* < 0.0001) (Figure 1I), and medicarpin (64 to 256 μg/mL|**** *p* < 0.0001) (Figure 1J) were significantly more effective to control parasite growth.

Based on the results from cell viability and *T. gondii* intracellular proliferation, we calculated the CC_50_, IC_50_, and SI (Table 1). BRP exhibited a CC_50_ > 256 µg/mL and an IC_50_ of 52.84 ± 6.31 µg/mL, resulting in an SI > 4.85. Vestitol showed a CC_50_ of 88.99 ± 14.55 µg/mL and an IC_50_ of 17.29 ± 0.17 µg/mL, with an SI of 5.15. 7-O-methylvestitol had a CC_50_ > 256 µg/mL and an IC_50_ of 22.83 ± 4.83 µg/mL, yielding the highest SI of 11.21. Neovestitol presented a CC_50_ of 73.45 ± 4.04 µg/mL, IC_50_ of 12.76 ± 0.70 µg/mL, and SI of 5.76. Lastly, medicarpin demonstrated a CC_50_ of 71.21 ± 2.49 µg/mL, IC_50_ of 9.46 ± 0.61 µg/mL, and an SI of 7.53. Based on their high selectivity indices and low cytotoxicity, BRP and 7-O-methylvestitol were selected for further assays in the present study. The concentration of 64 µg/mL was chosen for further experiments, as it represents an intermediate dose that effectively reduces *T. gondii* proliferation without showing cytotoxicity to host cells, thus representing a therapeutically relevant and safe concentration.

### 3.2. BRP and 7-O-methylvestitol Reduce T. gondii Intracellular Proliferation in an Irreversible Manner, Affecting Its Morphology

**Figure 2:**
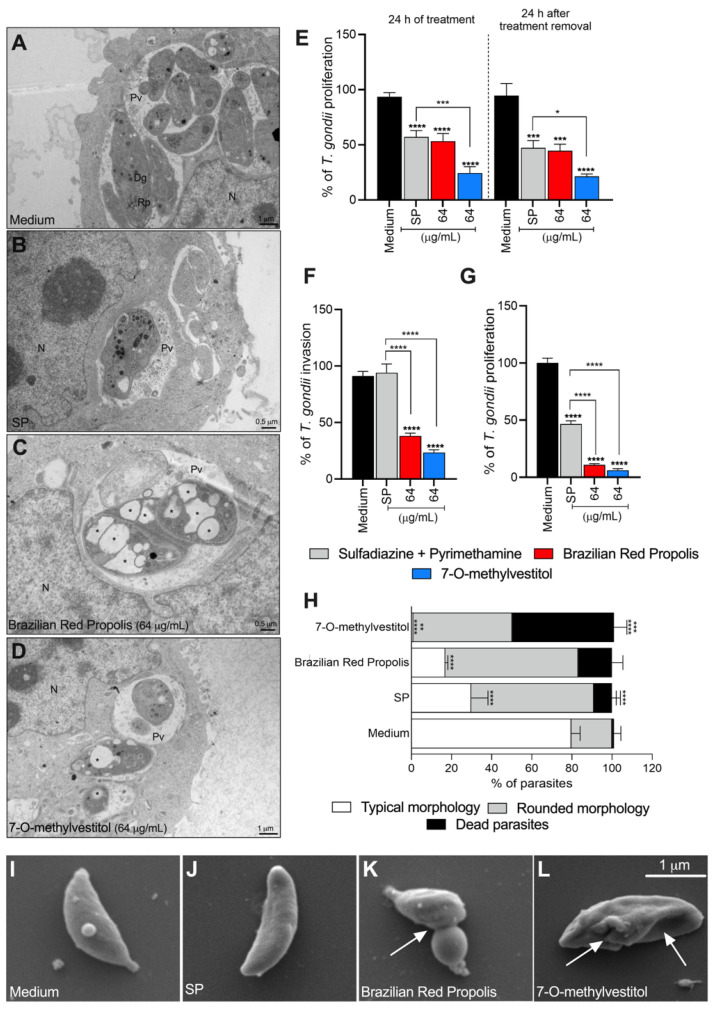
BRP and 7-O-methylvestitol irreversibly inhibit *T. gondii* intracellular proliferation while inducing morphological alterations in the parasite. BeWo cells were infected with *T. gondii* tachyzoites (MOI of 3:1) for 3 h and incubated for 24 h with BRP (64 µg/mL), 7-O-methylvestitol (64 µg/mL), SP (200 + 8 µg/mL, respectively), or culture medium only (untreated group). Representative transmission electron micrographs are demonstrated, according to the experimental situation: (**A**) untreated cells, (**B**) SP-treated cells, (**C**) BRP-treated cells, and (**D**) 7-O-methylvestitol-treated cells. Scale bars (bottom right): 0.5 and 1 μm. (**E**) Infected BeWo cells were treated for 24 h with BRP (64 µg/mL), 7-O-methylvestitol (64 µg/mL), SP (200 + 8 µg/mL), or culture medium only; in parallel, the same treatments were removed from infected cells, which were then maintained in a treatment-free medium for an additional 24 h. In both experimental conditions, intracellular parasite proliferation was assessed using the β-galactosidase assay. The reversibility assay evaluates the parasites’ capacity to recover from treatment and regain infectivity. *T. gondii* tachyzoites obtained from BeWo cells treated with BRP and 7-O-methylvestitol for 24 h were harvested and subsequently used to infect new BeWo cells for 3 h, to assess the invasion (**F**), or for 24 h to assess the proliferation (**G**). The number of tachyzoites was quantified using the β-galactosidase assay and expressed as a percentage of *T. gondii* invasion/proliferation. (**H**) *T. gondii* tachyzoites were treated for 1 h with BRP (64 µg/mL), 7-O-methylvestitol (64 µg/mL), SP (200 + 8 µg/mL), or culture medium only, and scanning electron microscopy was performed. Number of parasites with typical morphology, rounded morphology, or dead. Representative images are demonstrated, according to the experimental situation: (**I**) untreated group, (**J**) SP-treated parasites, (**K**) BRP-treated parasites, and (**L**) 7-O-methylvestitol-treated parasites. Scale bars: 1 μm. Data are shown as means ± standard error of the mean (SEM). Asterisks without brackets indicate comparisons versus the control group (black bar). Asterisks with brackets indicate comparisons between experimental groups. Significant differences were analyzed using a one-way ANOVA test with Sidak’s multiple comparison posttest. Differences were considered statistically significant when *p* < 0.05. Dg, dense granule; N, host nucleus; PV, parasitophorous vacuole; and Rp, rhoptries. White arrows indicate ultrastructural alterations.

To assess whether the treatments could target intracellular tachyzoites, we treated *T. gondii*-infected BeWo cells with BRP (64 µg/mL) or 7-O-methylvestitol (64 µg/mL) for 24 h, and the ultrastructure of the parasites and host cells was analyzed by transmission electron microscopy (TEM). According to the obtained images, the control group (infected but untreated cells) displayed several parasitophorous vacuoles (PVs) containing parasites with typical morphological characteristics, including roptries (Rp) and dense granules (Dg) near the nucleus (N) of the host cell (Figure 2A). As expected, SP treatment resulted in the disruption of parasite organelles, ruffled duple membranes, as well as parasites that appeared to have difficulty with the endogeny process (Figure 2B). BRP and 7-O-methylvestitol treatments induced morphological alterations in the parasites, making them rounder and containing vacuole-like structures inside (black asterisks) while also limiting intracellular proliferation compared to the control (Figure 2C,D). Both treatments induced marked disorganization and possible rupture of intracellular organelles in the parasites.

To determine whether the antiparasitic effects promoted by the compounds were transient or irreversible, *T. gondii*-infected BeWo cells were treated for 24 h. Afterward, the treatments were removed, and fresh culture medium was added to the cells for an additional 24 h. Intracellular proliferation was assessed using a β-galactosidase assay. As previously demonstrated, BRP, 7-O-methylvestitol (64 µg/mL|*** *p* < 0.001, **** *p* < 0.0001, respectively) and SP (**** *p* < 0.0001; Figure 2E) significantly controlled parasite proliferation compared to the control group for 24 h; in addition, 7-O-methylvestitol treatment was more effective than SP treatment (* *p* < 0.05; Figure 2E). Our results showed that 24 h after treatment removal, BRP, 7-O-methylvestitol, and SP (*** *p* < 0.001 and **** *p* < 0.0001) maintained their antiparasitic activity compared with the control group (infected/untreated cells) (Figure 2E). Interestingly, when comparing the condition 24 h after treatment removal with the corresponding 24 h treatment condition, no recovery of *T. gondii* proliferation was observed, indicating an irreversible antiparasitic effect (Figure 2E).

We further evaluated the invasion and proliferation capacities of *T. gondii* after 24 h of treatment with BRP or 7-O-methylvestitol. The results showed that both compounds effectively inhibited the parasite’s ability to invade (**** *p* < 0.0001; Figure 2F) and proliferate (**** *p* < 0.0001; Figure 2G) in freshly seeded BeWo cells in comparison with parasites obtained from infected/untreated cells (control group). Similarly, treatments were more effective at reducing the parasite load when compared to the conventional SP treatment (**** *p* < 0.0001; Figure 2F,G). As expected, SP treatment negatively affected only *T. gondii* intracellular proliferation (**** *p* < 0.0001; Figure 2G).

These data suggest that the mechanism of action of both compounds directly targets *T. gondii*. Subsequently, we assessed the viability of free tachyzoites treated for 1 h with the compounds using two techniques: trypan blue exclusion cell counting and scanning electron microscopy (SEM). Parasites exposed only to culture medium showed 80% of typical morphology (Figure 2H). Conventional SP treatment significantly reduced the proportion of parasites with typical morphology to approximately 30% (**** *p* < 0.0001) and increased the number of rounded parasites (**** *p* < 0.0001) (Figure 2H) when compared to the control group (untreated free tachyzoites). BRP (64 µg/mL) treatment resulted in approximately 60% (**** *p* < 0.0001) of parasites with a rounded morphology and less than 20% (**** *p* < 0.0001) with typical morphology in comparison to the control group (**** *p* < 0.0001) (Figure 2H). Regarding 7-O-methylvestitol treatment, an almost negligible percentage of parasites displayed typical morphology (**** *p* < 0.0001), with about 50% of parasites showing a rounded shape (**** *p* < 0.0001) and 50% being dead (**** *p* < 0.0001) (Figure 2H). Finally, 7-O-methylvestitol presented a higher proportion of dead (*** *p* < 0.001) and rounded morphology parasites (** *p* < 0.01) than SP treatment (Figure 2H). SEM analysis partially supported these findings, where untreated parasites (Figure 2I) and those treated with SP (Figure 2J) exhibited the typical crescent-shaped morphology. Meanwhile, parasites exposed to BRP (Figure 2K) and 7-O-methylvestitol (Figure 2L) showed clear surface damage (indicated by white arrows), swelling, and deformation, confirming the disruptive morphological effects induced by these treatments.

### 3.3. BRP and 7-O-methylvestitol Interfere with the Early Stages of T. gondii Infection in BeWo Cells

**Figure 3:**
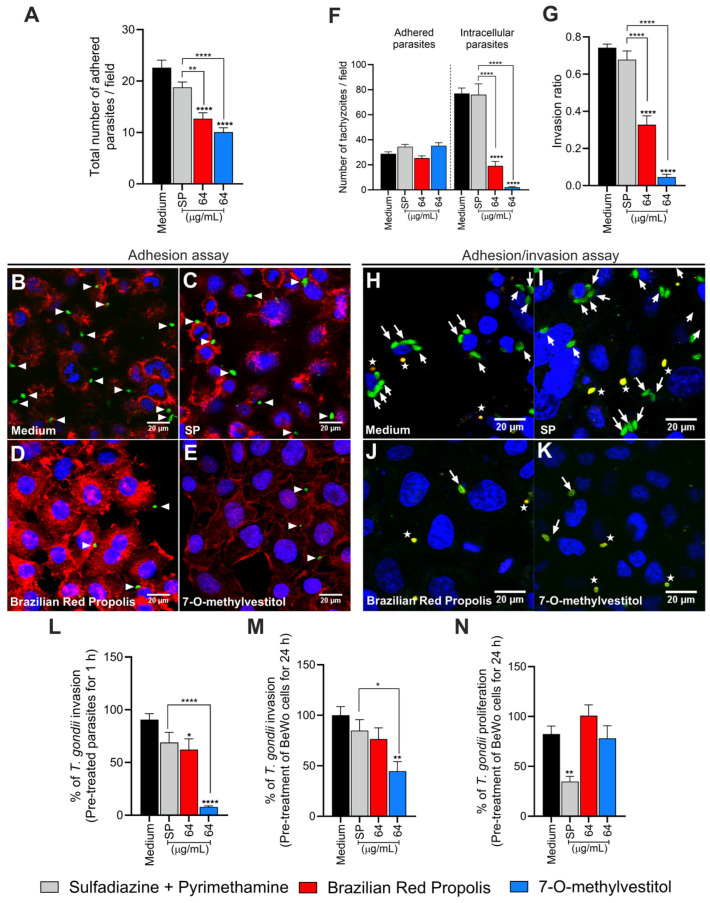
BRP and 7-O-methylvestitol interfere in early steps of *T. gondii* infection in BeWo cells. (**A**) *T. gondii* tachyzoites were pre-treated for 1 h with BRP (64 µg/mL), 7-O-methylvestitol (64 µg/mL), SP (200 + 8 µg/mL), or culture medium only, and then allowed to interact with paraformaldehyde-fixed BeWo cells on glass coverslips for 3 h. Subsequently, the parasites, actin filaments, and cell nuclei were stained, and the immunofluorescence assay was performed, and the number of adhered parasites was scored on 20 randomly selected fields. Representative fluorescence images are demonstrated according to the experimental situation: (**B**) untreated parasites, (**C**) SP-treated parasites, (**D**) BRP-treated parasites, and (**E**) 7-O-methylvestitol-treated parasites. (**B**–**E**) White arrowheads indicate adhered parasites. (**F**,**G**) *T. gondii* tachyzoites were pre-treated as described above and then used to infect BeWo cells on glass coverslips for 3 h. After, the parasites and cell nuclei were stained for immunofluorescence reaction. The numbers of adhered [red or red^+^/green^+^ (yellow)] and intracellular (green^+^/red^−^) parasites were scored on 20 randomly selected fields. The proportion of the number of intracellular tachyzoites to the total number of parasites was considered the invasion ratio. Representative fluorescence images are demonstrated according to the experimental situation: (**H**) untreated parasites, (**I**) SP-treated parasites, (**J**) BRP-treated parasites, and (**K**) 7-O-methylvestitol-treated parasites. (**H**–**K**) White arrows indicate intracellular parasites, while white asterisks mark parasites adhered to the cell surface. (**L**) *T. gondii* tachyzoites were pre-treated as described above and then used to infect BeWo cells for 3 h. The number of tachyzoites was quantified using the β-galactosidase assay and expressed as a percentage of *T. gondii* invasion. (**M**,**N**) BeWo cells were pre-treated for 24 h with BRP (64 µg/mL), 7-O-methylvestitol (64 µg/mL), SP (200 + 8 µg/mL), or culture medium only. Then, the cells were infected with *T. gondii* tachyzoites (MOI of 3:1) for 3 or 24 h to assess the invasion and proliferation, respectively. The number of tachyzoites was quantified using the β-galactosidase assay and expressed as a percentage of *T. gondii* invasion/proliferation. Data are shown as means ± standard error of the mean (SEM). Asterisks without brackets indicate comparisons versus the control group (black bar). Asterisks with brackets indicate comparisons between experimental groups. Significant differences were analyzed using one-way ANOVA test with Sidak’s multiple comparison posttest. Differences were considered statistically significant when *p* < 0.05. White arrowheads indicate parasites adhered to the cells. White arrows indicate intracellular parasites. White asterisks indicate adhered parasites. Scale bars: 20 μm.

To further investigate the potential targets of BRP and 7-O-methylvestitol, *T. gondii* tachyzoites were pre-treated for 1 h, and the adhesion assay was performed. When BeWo cells were incubated with tachyzoites pre-treated with BRP or 7-O-methylvestitol (both 64 µg/mL), a lower number of adhered parasites was observed compared to the control group (**** *p* < 0.0001) and the SP-treated group (** *p* < 0.01 and **** *p* < 0.0001, respectively) (Figure 3A–E). SP treatment did not show a significant difference in the number of adhered parasites compared to the control (Figure 3A,C). Using the green–red assay, we found that pre-treatment with BRP or 7-O-methylvestitol (both 64 µg/mL) did not affect the number of adhered parasites compared to the control (Figure 3F). However, BRP and 7-O-methylvestitol diminished the number of intracellular parasites (**** *p* < 0.0001; Figure 3F) and invasion ratio (**** *p* < 0.0001; Figure 3G). Interestingly, the antiparasitic effects of BRP and 7-O-methylvestitol were stronger than those of the classical SP treatment (**** *p* < 0.0001; Figure 3F,G). Illustrative fluorescence images are demonstrated (Figure 3H–K).

The reduced parasitic invasion rate following pre-treatment of parasites for 1 h with BRP or 7-O-methylvestitol was confirmed by the β-galactosidase assay. Parasites pre-treated with BRP exhibited lower parasitic invasion percentage compared to the control (* *p* < 0.05; Figure 3L), while the pre-treatment with 7-O-methylvestitol reduced invasion compared to both the control and SP-treated group (**** *p* < 0.0001) (Figure 3L). In contrast, SP treatment did not alter the invasion ratio compared to the control (Figure 3L). These results indicate that BRP and 7-O-methylvestitol are capable of interfering with the early stages of *T. gondii* infection in BeWo cells and corroborate the TEM and SEM data, demonstrating that these compounds have a direct action on the parasite.

To investigate whether the compounds could target the host cell, we pre-treated BeWo cells for 24 h and subsequently performed invasion and proliferation assays. Our results showed that BRP treatment did not affect either *T. gondii* invasion or proliferation compared to the control group (Figure 3M,N). On the other hand, 7-O-methylvestitol treatment reduced invasion compared to the control and SP-treated groups (** *p* < 0.01 and * *p* < 0.05, respectively) (Figure 3M) but did not affect parasite proliferation (Figure 3N). Pre-treatment of BeWo cells with SP did not affect the invasion process (Figure 3M) but significantly restricted intracellular parasite growth compared to untreated cells (control group) (** *p* < 0.01; Figure 3N).

### 3.4. BRP and 7-O-methylvestitol Increase IL-6, IL-8, and MIF Levels and Alter ROS Production in BeWo Cells

To evaluate the immunomodulatory capacity of BRP and 7-O-methylvestitol in BeWo cells infected or not with *T. gondii*, we quantified the cytokines IL-6, IL-8, IL-10, MIF, and TNF.

**Figure 4:**
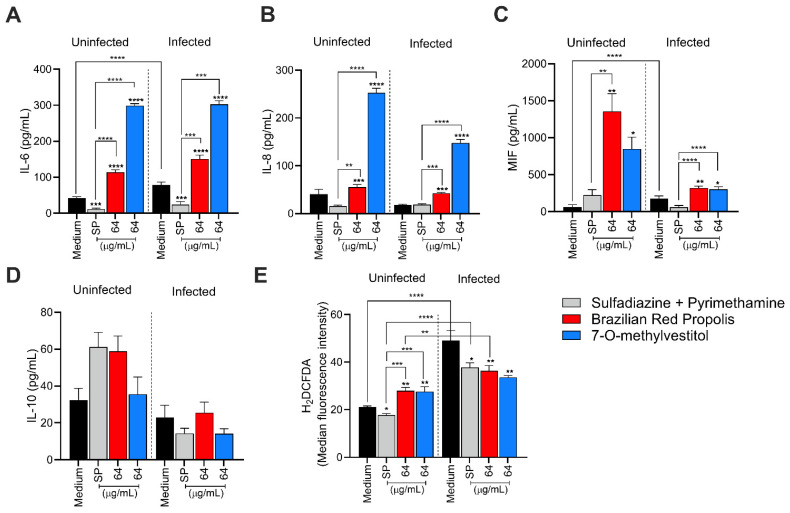
BRP and 7-O-methylvestitol lead to an increase in IL-6, IL-8, and MIF levels and alter ROS production in BeWo cells. BeWo cells were infected or not with *T. gondii* tachyzoites, followed by treatment for 24 h with BRP (64 µg/mL), 7-O-methylvestitol (64 µg/mL), SP (200 + 8 µg/mL), or culture medium only. Cell culture supernatants were collected for measurement of (**A**) IL-6, (**B**) IL-8, (**C**) MIF, and (**D**) IL-10. Cytokine levels were expressed in pg/mL. (**E**) The fluorescent probe H_2_DCF-DA was used to measure the production of ROS in the BeWo cells. Data are shown as means ± standard error of the mean (SEM). Asterisks without brackets indicate comparisons versus the control group (black bar). Asterisks with brackets indicate comparisons between experimental groups. Significant differences were analyzed using one-way ANOVA test with Sidak’s multiple comparison posttest. Differences were considered statistically significant when *p* < 0.05.

In the absence of infection, treatments with BRP and 7-O-methylvestitol (both 64 μg/mL) increased IL-6 production compared to the untreated control and conventional treatment (**** *p* < 0.0001) (Figure 4A). Infection by *T. gondii* increased IL-6 levels in the control group compared to uninfected/untreated cells (**** *p* < 0.0001). The groups treated with BRP and its isolated compound, maintained high IL-6 levels observed, even in the presence of parasitic infection compared to the infected/untreated BeWo cells (**** *p* < 0.0001); in contrast, SP-treated/infected BeWo cells downregulated IL-6 both in the absence and presence of *T. gondii* in relation to the untreated groups (*** *p* < 0.001) (Figure 4A).

Regarding IL-8, uninfected cells treated with BRP or 7-O-methylvestitol revealed higher cytokine production compared to both the uninfected/untreated control (*** *p* < 0.001 and **** *p* < 0.0001, respectively) and the uninfected/SP-treated group (** *p* < 0.01 and **** *p* < 0.0001, respectively) (Figure 4B). Similarly, in the presence of *T. gondii* infection, BRP or 7-O-methylvestitol increased IL-8 production in relation to both infected/untreated control (*** *p* < 0.001 and **** *p* < 0.0001, respectively) and the infected/SP-treated group (*** *p* < 0.001 and **** *p* < 0.0001, respectively) (Figure 4B).

In the absence of *T. gondii*, BRP or 7-O-methylvestitol treatments upregulated MIF production compared to the untreated control (** *p* < 0.01 and * *p* < 0.05, respectively), and only BRP-treated BeWo cells demonstrated higher MIF levels than the SP-treated group (** *p* < 0.01; Figure 4C). *T. gondii* infection increased MIF production compared to uninfected/untreated cells (**** *p* < 0.0001); both BRP and its isolated compound augmented MIF levels compared to the infected/untreated control (** *p* < 0.01 and * *p* < 0.05, respectively) and SP treatment (**** *p* < 0.0001) (Figure 4C). The production of IL-10 and TNF was also evaluated in BeWo cells, but the TNF levels were below the detection limit, and IL-10 levels did not present significant differences among the experimental groups (Figure 4D).

Regarding ROS production, in the absence of infection, BeWo cells treated with BRP and 7-O-methylvestitol showed higher ROS production compared to both the uninfected control (** *p* < 0.01) and the SP-treated group (*** *p* < 0.001); in addition, cells treated with SP exhibited lower ROS production compared to the uninfected/untreated control (* *p* < 0.05) (Figure 4E). *T. gondii* infection increased ROS levels in BeWo cells compared to the uninfected/untreated group (**** *p* < 0.0001; Figure 4E). Infected cells exposed to treatment with SP or BRP showed lower ROS production compared to the infected/untreated group (* *p* < 0.05 and ** *p* < 0.01, respectively), but higher levels compared to their respective uninfected groups (**** *p* < 0.0001 and ** *p* < 0.01, respectively); in contrast, infected cells treated with 7-O-methylvestitol exhibited lower ROS production compared to the positive control (** *p* < 0.01), but no statistical difference was observed compared to the respective treatment in the absence of infection (Figure 4E). Thus, we conclude that BRP and 7-O-methylvestitol play a modulatory role in the cytokine profile, primarily by increasing the secretion of IL-6, IL-8, and MIF, as well as modulating ROS production in BeWo cells.

### 3.5. BRP and 7-O-methylvestitol Control T. gondii Proliferation in Human Placental Explants and Downregulate IL-6, MIF, and TNF Production

**Figure 5:**
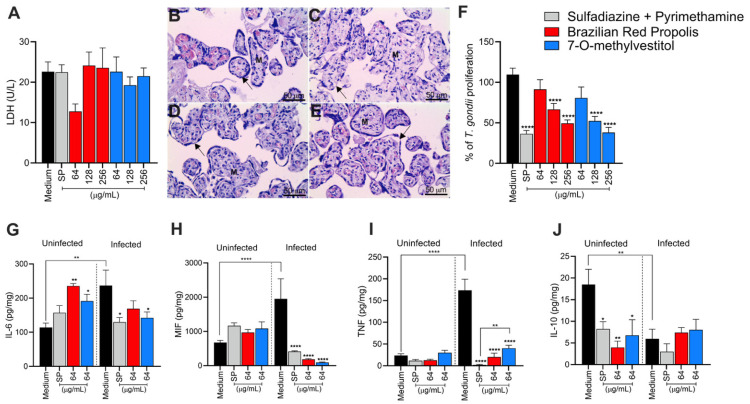
BRP and 7-O-methylvestitol inhibit *T. gondii* proliferation in human placental explants and modulate cytokine production. Villous explants were incubated for 24 h with BRP (128 µg/mL), 7-O-methylvestitol (128 µg/mL), SP (200 + 8 µg/mL), or culture medium only, and subsequently assessed for viability. (**A**) Collected villous supernatants were analyzed to determine LDH levels (U/L). Histological sections were stained with hematoxylin–eosin (HE). Representative photomicrographs of tissues are demonstrated according to the experimental situation: (**B**) untreated villous, (**C**) SP-treated villous, (**D**) BRP-treated villous, and (**E**) 7-O-methylvestitol-treated villous. (**F**) Villous explants were infected with *T. gondii* tachyzoites for 24 h, followed by treatment for additional 24 h with BRP (128 µg/mL), 7-O-methylvestitol (128 µg/mL), SP (150 + 200 μg/mL), or culture medium only. Intracellular parasite proliferation was assessed using the β-galactosidase assay and represented as percentage *T. gondii* proliferation, with the untreated/infected group (control) considered as 100% of parasite proliferation. Supernatant from uninfected or infected villous after treatments was collected for measurement of (**G**) IL-6, (**H**) MIF, (**I**) TNF, and (**J**) IL-10. Cytokine levels were expressed in pg/mg of tissue. Data are shown as means ± standard error of the mean (SEM). Asterisks without brackets indicate comparisons versus the control group (black bar). Asterisks with brackets indicate comparisons between experimental groups. Significant differences were analyzed using one-way ANOVA test with Sidak’s multiple comparison posttest. Differences were considered statistically significant when *p* < 0.05. Black arrows indicate syncytiotrophoblasts, and “M” indicates mesenchyme. Scale bars: 50 μm.

Ex vivo models of human placental explants have been widely used in studies involving *T. gondii* infection at the maternal–fetal interface. Thus, to corroborate the results obtained from assays with BeWo cells, we also investigated the potential of BRP and 7-O-methylvestitol to control *T. gondii* infection in placental villi. First, we assessed the viability of explants exposed to treatment with the compounds at three concentrations (64, 128, and 256 µg/mL) and observed no significant difference in LDH levels released compared to the control group (untreated villous) (Figure 5A). To reinforce the biochemical findings, we performed hematoxylin–eosin (HE) staining to conduct a morphological analysis and assess the integrity of the chorionic villi comprising the placental tissue. We observed that tissues treated with the highest concentration of BRP or 7-O-methylvestitol (256 µg/mL) exhibited morphological characteristics identical to those observed in the untreated control, such as a continuous layer of syncytiotrophoblasts (indicated by black arrows) covering the mesenchyme (M) (Figure 5B–E). After confirming that both compounds are non-toxic to placental villi, we evaluated their effects on *T. gondii* proliferation in the tissue. The results showed that at concentrations of 128 and 256 µg/mL, BRP and 7-O-methylvestitol significantly reduced the parasite load compared to the untreated control (**** *p* < 0.0001; Figure 5F). SP was used as a positive control and significantly reduced parasite proliferation compared to the untreated control (**** *p* < 0.0001; Figure 5F).

Additionally, we evaluated cytokine production in explants exposed to treatments at the concentration of 128 µg/mL, a concentration chosen for its intermediate level, safety, and efficacy. Regarding IL-6 production, in the absence of the parasite, treatment with BRP or 7-O-methylvestitol promoted an increase in cytokine levels compared to the control (** *p* < 0.01 and * *p* < 0.05, respectively) (Figure 5G). Meanwhile, *T. gondii* infection led to an increase in IL-6 in the untreated control compared to the uninfected control (** *p* < 0.01); however, treatments with 7-O-methylvestitol and SP decreased these cytokine levels compared to the infected/untreated group (* *p* < 0.05) (Figure 5G). Regarding MIF, levels remained similar between groups in the absence of the parasite (Figure 5H), but with *T. gondii* infection, the control group exhibited an accentuated increase (**** *p* < 0.0001) in comparison with the uninfected/untreated group, which was significantly reduced by all treatments (**** *p* < 0.0001) (Figure 5H). TNF showed a similar behavior, with low levels in the uninfected groups and an increase in the infected control (**** *p* < 0.0001; Figure 5I) in relation to the uninfected/untreated group, while the treatments demonstrated great efficacy in reducing these levels (**** *p* < 0.0001; Figure 5I). In addition, only 7-O-methylvestitol treatment maintained a slightly higher TNF induction compared to the infected/SP-treated group (** *p* < 0.01; Figure 5I). For IL-10, the uninfected control showed higher levels (Figure 5J), while treatments with SP (* *p* < 0.05), BRP (** *p* < 0.01), and 7-O-methylvestitol (* *p* < 0.05) reduced these values. Following parasite infection, IL-10 levels decreased in the untreated group compared to the uninfected/untreated control (** *p* < 0.01) and remained unchanged in the treated groups (Figure 5J). No significant differences were observed in IL-8 production among the experimental groups analyzed.

## 4. Discussion

As mentioned before, Propolis is widely recognized as a valuable source of natural antioxidant compounds, whose composition may vary due to several factors, such as seasonality, light exposure, altitude, local flora, and the bee species responsible for collection [12,13]. According to Freires et al. (2016) [12], Brazilian Red Propolis contains a variety of important compounds, including isoflavonoids, pterocarpans, chalcones, flavonoids, prenylated benzophenones, terpenes, and tannins, which have been associated with antiparasitic and immunomodulatory activities. In line with the findings by Machado et al. (2016) and Aldana-Mejía et al. (2025) [13,25], our data confirm the broad spectrum of bioactive compounds present in Brazilian Red Propolis, further supporting its potential as an effective therapeutic agent in parasite control and immune modulation.

Our results demonstrated that cells treated with BRP and its isolated compounds, including 7-O-methylvestitol, did not affect viability at any of the tested concentrations. This observation is consistent with Aldana-Mejía et al. (2025) [25], who reported that these compounds do not exhibit cytotoxic activity in VERO and LLC-PK1 cells, emphasizing their safe profile for therapeutic use. Furthermore, these compounds effectively controlled *T. gondii* intracellular proliferation, showing comparable efficacy to the conventional treatment with SP or, in some concentrations, a better potential than SP. In addition, we calculated the selectivity index (SI) and observed that all compounds in our present study, especially 7-O-methylvestitol, presented their therapeutic potential by demonstrating their selectivity in targeting the parasite without adversely affecting host cell viability, reinforcing their promise as antiparasitic agents. Several studies have highlighted the antiparasitic potential of propolis against pathogenic protozoa. For instance, Regueira-Neto et al. (2018) [11] reported that Brazilian Red Propolis significantly inhibited the proliferation of *Leishmania braziliensis* and *L. infantum* promastigotes, as well as *Trypanosoma cruzi* epimastigotes of the CL-B5 strain. Also, Brazilian Amazon red propolis exhibited an inhibitory activity against *L. amazonensis* and its isolated compounds, especially flavonoids, showed potential to reduce the promastigote replication and presented high binding affinity to targets in this specific parasite [26]. Furthermore, 7-O-methylvestitol has previously been shown to possess antimalarial properties [25], and ethanolic extracts of propolis presented the ability to minimize the growth of *Cryptosporidium* spp., *Giardia lamblia*, *Trichomonas vaginalis,* and *Blastocystis* spp. [27]. The effect of propolis is still observed against helminths since some studies evidenced anti-*Echinococcus*, anti-*Fasciola*, anti-*Schistosoma,* and anti-*Trichinella* activity of several sources of propolis worldwide [28]. Finally, regarding *T. gondii*, there is only one recent study showing that Guttiferone E and Oblongifolin B, compounds also isolated from Brazilian Red Propolis, inhibited *T. gondii* growth in BeWo cells, but the mechanisms of action were not determined [29].

We investigated the mechanisms by which the compounds act against *T. gondii* and observed that treatment with BRP and 7-O-methylvestitol caused significant damage to the internal morphology of *T. gondii*, leading to cytoplasmic disorganization, loss of plasma membrane integrity, and vacuolization. The external morphology was also compromised, resulting in rounded and dead parasites. This finding is consistent with previous observations, when significant morphological alterations were detected in the tegument of *Schistosoma mansoni* treated with BRP, reinforcing the efficacy of these compounds in parasite disintegration [30]. Also, trypanocidal activity of ethanolic extract of different propolis sources, such as Brazil (Et-Bra) and Bulgaria (Et-Blg), was significantly associated with potential damage in the ultrastructure of epimastigotes and amastigotes. The treatment of trypomastigotes with Et-Blg promoted mitochondrial alterations, and Et-Bra triggered damage to plasma membranes. Epimastigotes treated with both extracts presented mitochondrial swelling, while Et-Bra-treated parasites demonstrated disruption of the Golgi complex [31]. Thus, it is widely known that propolis is able to affect directly the morphology and ultrastructure in different parasites, but this study is the first to show the damages caused by BRP and 7-O-methylvestitol in *T. gondii* tachyzoites and how it can influence in the several stages of infection of this parasite since the reversibility assay supported the hypothesis that the damage is irreversible. Furthermore, parasites derived from BeWo cells treated with BRP or 7-O-methylvestitol presented compromised reinfection ability, suggesting one more time that the damage to ultrastructure was essential to impair the infection propagation. These compounds target essential cellular components of the parasite, such as membranes, organelles, or metabolic pathways, configuring an important attribute for potential therapies, as it minimizes the risk of reinfection or resistance. Similar findings on irreversibility have recently been demonstrated by our group in previous studies using other compounds [20].

After verifying the potent effects of BRP and 7-O-methylvestitol in controlling intracellular parasite proliferation and potentially preventing its propagation irreversibly, as well as detecting severe damage in tachyzoites, we became interested in investigating the initial steps of *T. gondii* infection—particularly adhesion and invasion—using BeWo cells or parasites previously treated with BRP or 7-O-methylvestitol. As a result, we could observe that pre-treated parasites presented difficulty in adhering and invading BeWo cells. The reduction in parasite adhesion and invasion after just one hour of treatment reinforces that these compounds act directly on the parasite, as previously detected by SEM, and/or on molecular components involved in the host-parasite interaction that morphological assays are unable to detect. Furthermore, the compounds proved to be even more effective than conventional treatment, indicating that they may alter specific adhesion factors such as surface proteins or other cellular components of *T. gondii*. This aligns with a previous study in which vestitol, an isolated component of Brazilian Red Propolis, exhibited activity on the plasma membrane of *T. cruzi* as an antiparasitic mechanism [32]. Additionally, the antimycotic activity of propolis collected from two Mexican regions was associated with the destruction of the cell wall and plasma membrane of fungi [33]. In the next step, our findings verified that the pre-treatment of BeWo cells with BRP and 7-O-methylvestitol also impaired the invasion and replication rates of *T. gondii*, suggesting that these respective extracts and compound have the ability to modulate the host cell in addition to their own parasite. There are some studies in the literature showing that propolis extracts are able to reduce the intercellular adhesion molecule (ICAM)-1 expression in several host cells as human fibroblasts [34], mouse aortic endothelial cells [35], and peritoneal macrophages [36]. Neovestitol, one of the isoflavonoids isolated from Brazilian Red Propolis used in this present study, presented the ability to diminish the acute peritonitis induced by LPS in mice, and this effect was associated with ICAM-1 downmodulation [10]. Our previous studies demonstrated that ICAM-1 is an important molecule present in BeWo cells used by *T. gondii* tachyzoites to adhere and invade these host cells, evidencing an important gateway to infection in the maternal–fetal interface [21]. Therefore, we can hypothesize that BRP and 7-O-methylvestitol can downmodulate ICAM-1 in pre-treated BeWo cells and consequently harm the adhesion and invasion of *T. gondii*. Future studies are necessary to investigate the effect of BRP and 7-O-methylvestitol on the expression of ICAM-1 in human trophoblast cells.

Several studies from our group have linked cytokine modulation to the susceptibility of trophoblastic cells to *T. gondii* [22]. In this sense, we investigated how BRP and 7-O-methylvestitol modulate these immune mediators in BeWo cells and their impact on parasite infection. Our results show that BRP and 7-O-methylvestitol triggered a modulatory role in the cytokine profile, primarily by increasing the secretion of IL-6, IL-8, and MIF, as well as downmodulating ROS production in infected BeWo cells. The augment of IL-6 and MIF induced by BRP and 7-O-methylvestitol plays a relevant role in controlling the *T. gondii* proliferation since both cytokines are widely known to reduce the infections in several cell types, including trophoblast [30]. Thus, the upregulation of MIF and IL-6, aligned to damage in tachyzoites’ structure, works as a potent and efficient anti-*T. gondii* activity induced by BRP and 7-O-methylvestitol in BeWo cells. The role of IL-8 in maternal–fetal interface is still unclear; however, our previous studies have demonstrated that this immune mediator was relevant in favor indirectly the parasite growth in human extravillous trophoblast cells [22]; then, we cannot establish a relation between controlled *T. gondii* infection and IL-8 upregulation triggered by BRP and 7-O-methylvestitol, although other studies display an efficient recruitment of innate immune cells induced by IL-8 in a scenario of *T. gondii* infection, suggesting the protector role of IL-8 against parasite dissemination [37,38]. Although no statistically significant difference was observed for IL-10, BRP and 7-O-methylvestitol modulated ROS production both in the absence and presence of *T. gondii*. In the presence of the parasite, infection only induced a significant increase in ROS levels, highlighting the activation of the immune system in response to infection. However, when treatments with BRP and 7-O-methylvestitol were included, ROS levels were downmodulated, which may suggest a regulatory role of these compounds in controlling oxidative stress caused by the parasite and a potential ability to balance the immune response against *T. gondii*. The activation of inflammasome complexes in response to cellular damage caused by *T. gondii* is well-documented in the literature and supports the findings of this study [39,40,41]. In conclusion, we can affirm that BRP and 7-O-methylvestitol reduced *T. gondii* infection in BeWo cells by mechanisms of action activated directly in parasites and host cell when trigger damages on tachyzoite and modulate the immune profile to combat the infection, respectively.

Although we have demonstrated that BRP and 7-O-methylvestitol induced proinflammatory cytokines in BeWo cells, several studies in the literature declare propolis and its isolates as inductors of regulatory and anti-inflammatory responses. Propolis was able to inhibit several types of proinflammatory mediators as TLR4, MyD88, IRAK4, TRIF, NLRP inflammasomes, NF-κB, and their associated cytokines, such as IL-1β, IL-6, IFN-γ, and TNF-α [42]. In addition, different propolis sources prevented LPS-induced IL-17A production and promoted Th2 activation in CD4+ T cells [43], while BRP activated downmodulation of IL-1α, IL-1β, IL-4, IL-6, IL-12p40, IL12-p70, IL-13, MCP1, and GM-CSF in peritoneal macrophages [28]. In vivo studies have already demonstrated the ability of propolis extracts to inhibit the increase in IL-6 in serum and inflamed tissues [44,45]. Interestingly, Brazilian propolis played a significant immunomodulatory role through inhibition of TNF-α, CXCL1/KC, and CXCL2/MIP2; ERK1/2, JNK, and p38MAPK phosphorylation; NF-κB activation; and ICAM-1, VCAM-1, and E-selectin expression [46]. In our present study, we verified a downmodulation of ROS induced by BRP and 7-O-methylvestitol, and it aligns with the known anti-inflammatory activity of propolis. Then, it seems that BRP and 7-O-methylvestitol still maintain a tolerant immune profile, at least partially, even inducing high levels of IL-6 and MIF.

To corroborate our in vitro data, we analyzed the ability of BRP and 7-O-methylvestitol to control *T. gondii* infection in an ex vivo model (third-trimester human villous explants). The LDH release assay reinforced the non-toxicity of the compounds and the absence of tissue damage, which was further confirmed by morphological analysis. In this analysis, we observed the preservation of placental tissue integrity in the groups treated with BRP and 7-O-methylvestitol, in contrast to the conventional treatment, which led to discontinuity of the syncytiotrophoblast layer. The significant reduction in parasite load, particularly at higher concentrations, highlights the antiparasitic potential of these compounds and supports the findings in BeWo cells. However, contrary to BeWo cells, BRP and 7-O-methylvestitol promoted a significant reduction in IL-6, MIF, and TNF levels, demonstrating their anti-inflammatory profile widely declared in literature [42,46]. It is important to emphasize that this regulatory profile is relevant to maintain the successful gestation [47,48]; thus, BRP and 7-O-methylvestitol are able to control Toxoplasma infection without harming the tolerogenic immune response necessary to embryonic or fetal development. Thus, it is possible to conclude that the control of *T. gondii* growth in human explants can be associated with only direct damage in the parasite instigated by both treatments. Future studies are necessary to investigate other mechanisms of action induced by Brazilian propolis in the human placenta in the context of toxoplasmosis.

Our findings suggest that the irreversible antiparasitic effects of BRP are likely associated with the action of its active component, especially 7-O-methylvestitol, which directly targets *T. gondii*, compromising both its external structure and internal organization. Additionally, we observed that the immune alterations induced by these compounds were crucial in controlling parasitism in BeWo cells. These results highlight the potential of 7-O-methylvestitol as a key bioactive agent within BRP, emphasizing its promise as an immunomodulatory compound capable of restoring immune balance in the maternal–fetal interface, effectively managing the infection, and preventing excessive immune dysregulation that could lead to placental tissue damage. Understanding the specific action of 7-O-methylvestitol could serve as a valuable tool in the design of new drugs and the identification of novel therapeutic targets for congenital toxoplasmosis.

## 5. Conclusions

This study demonstrated significant anti-*T. gondii* effects of BRP and 7-O-methylvestitol through both in vitro and ex vivo assays. BRP and 7-O-methylvestitol irreversibly controlled the infection with low toxicity to host cells and a direct effect on the parasite while efficiently modulating the immune response. These findings highlight the relevance of natural products such as BRP in the development of alternative therapies against parasitic infections, especially those associated with the maternal–fetal interface.

## Figures and Tables

**Table 1:** Structure and in vitro activity of the BRP and its isolated compounds against *T. gondii*.

Compounds	Chemical Structure	CC_50_ (μg/mL) ± SD	IC_50_ (μg/mL) ± SD	SI (CC_50_/IC_50_)
Brazilian Red Propolis (BRP)	-	>256	52.84 ± 6.31	>4.85
Vestitol	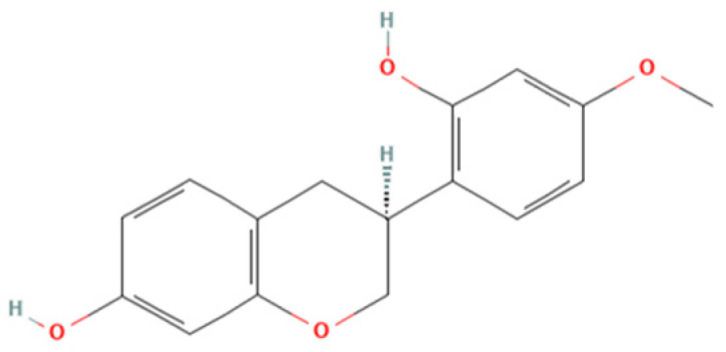	88.99 ± 14.55	17.29 ± 0.17	5.15
7-O-methylvestitol	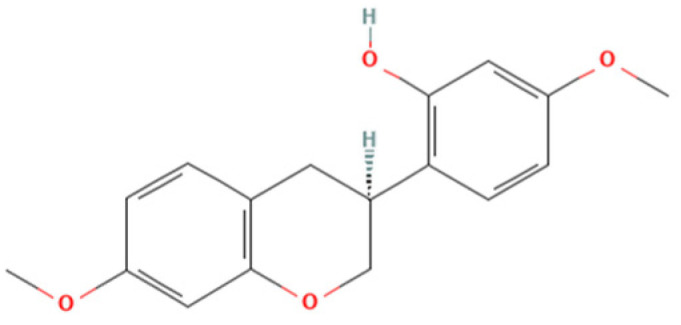	>256	22.83 ± 4.83	11.21
Neovestitol	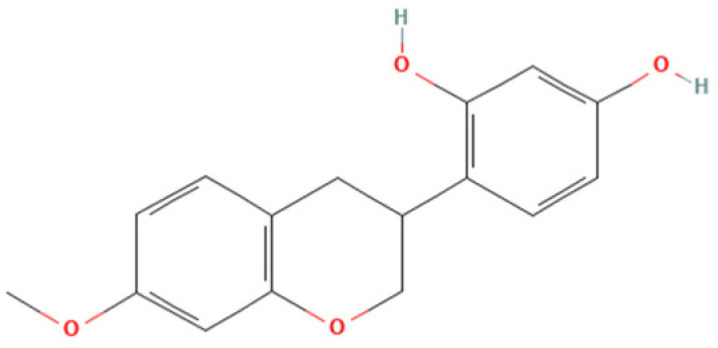	73.45 ± 4.035	12.76 ± 0.70	5.76
Medicarpin	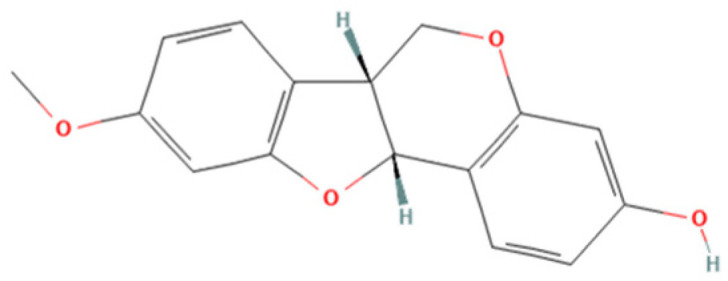	71.21 ± 2.49	9.46 ± 0.610	7.53

CC_50_ (50% cytotoxic concentration): the concentration of the compound that reduces host cell viability by 50%; IC_50_ (50% inhibitory concentration): the concentration of the compound that inhibits 50% of *T. gondii* proliferation; SI (selectivity index): the ratio between CC_50_ and IC_50_ values (SI = CC_50_/IC_50_), indicating the compound’s selectivity for the parasite over host cells.

## Data Availability

The original contributions presented in this study are included in the article. Further inquiries can be directed to the corresponding authors.
